# Altered synaptic and astrocytic proteins in Lewy body disorders associated with *GBA* mutations

**DOI:** 10.1093/braincomms/fcag246

**Published:** 2026-07-23

**Authors:** Thamir M Eid, Tammaryn Lashley, Thomas T Warner, Alaa Khan, Chanati Jantrachotechatchawan, Paul T Francis, John Hardy, Rina Bandopadhyay

**Affiliations:** Queen Square Brain Bank for Neurological Disorders, University College London Queen Square Institute of Neurology, London WC1N 1PJ, UK; Department of Clinical and Movement Neurosciences, University College London Queen Square Institute of Neurology, London WC1N 3BG, UK; Institute of Psychiatry, Psychology, and Neuroscience, King’s College London, London SE5 8AB, UK; Department of Biochemistry, Faculty of Science, King Abdulaziz University, Jeddah 21589, Saudi Arabia; Department of Neurodegenerative Disease, University College London Queen Square Institute of Neurology, London WC1N 3BG, UK; Queen Square Brain Bank for Neurological Disorders, University College London Queen Square Institute of Neurology, London WC1N 1PJ, UK; Department of Neurodegenerative Disease, University College London Queen Square Institute of Neurology, London WC1N 3BG, UK; Reta Lila Weston Institute, University College London Queen Square Institute of Neurology, London WC1N 1PJ, UK; Department of Neuromuscular Diseases, University College London Queen Square Institute of Neurology, University College London, London WC1N 3BG, UK; Institute of Psychiatry, Psychology, and Neuroscience, King’s College London, London SE5 8AB, UK; Research Department, Faculty of Medicine Siriraj Hospital, Mahidol University, Bangkok 10700, Thailand; Institute of Psychiatry, Psychology, and Neuroscience, King’s College London, London SE5 8AB, UK; Department of Neurodegenerative Disease, University College London Queen Square Institute of Neurology, London WC1N 3BG, UK; Department of Clinical and Movement Neurosciences, University College London Queen Square Institute of Neurology, London WC1N 3BG, UK; Reta Lila Weston Institute, University College London Queen Square Institute of Neurology, London WC1N 1PJ, UK

**Keywords:** Parkinson’s disease (PD), glucocerebrosidase (*GBA*) mutations, synaptic proteins, α-synuclein (α-syn), neurodegeneration

## Abstract

Mutations in the glucocerebrosidase (*GBA*) gene, originally implicated in Gaucher’s disease, are now recognized as a major risk factor for developing Parkinson’s disease (PD). While up to 70% of PD patients eventually progress to dementia, *GBA* mutations further increase the risk of Lewy body dementias (LBD), an umbrella term encompassing both dementia with Lewy bodies and Parkinson’s disease dementia. Collectively, these disorders are classified as synucleinopathies. To date, there is no clear understanding of the mechanistic relationships between *GBA* mutations and synucleinopathies, although, synaptic protein changes have been shown to correlate with cognitive change in LBD as well as in Alzheimer’s disease (AD). The aim of this study was to examine synaptic dysfunction in α-synucleinopathies with *GBA* mutations. The research cohort consisted of 10 controls, *7* PD/LBD-*GBA* N370S, and 20 PD/LBD-wild type (WT) where seven synaptic markers including four pre-synaptic [synaptosomal-associated protein 25; (SNAP25), synaptophysin; (SYP), ras-related protein; (Rab3A), and vesicle associated membrane proteins 2; (VAMP2)], two post-synaptic [post-synaptic density protein 95; (PSD95), and neurogranin; (NRGN)], and one astrocytic [vesicle associated membrane proteins 3; (VAMP3)] protein were investigated in four cortical regions (prefrontal, temporal, anterior cingulate and parietal) using immunoblot technique. Four markers (NRGN, SNAP25, VAMP2, and VAMP3) were found to be significantly and regionally altered between the three groups. The expression of pre-synaptic (SNAP25), post-synaptic (NRGN), and the astrocytic protein (VAMP3) is different between PD/LBD with *GBA* mutation and PD/LBD-WT suggesting the potential role of *GBA* gene due to mutations which could alter the levels of some synaptic markers. Our study is the first to validate several synaptic markers in human post-mortem-associated *GBA* N370S mutation.

## Teaser

Eid *et al*. investigated synaptic proteins in post-mortem brains of patients with Parkinson’s disease and Lewy body dementia, comparing those with GBA mutations to wild-type cases and controls. They found distinct region-specific alterations in synaptic markers like synaptosomal-associated protein 25 (SNAP25) and neurogranin (NRGN), suggesting that GBA mutations contribute to unique molecular pathologies.

## Graphical Abstract

**Graphical Abstract:**
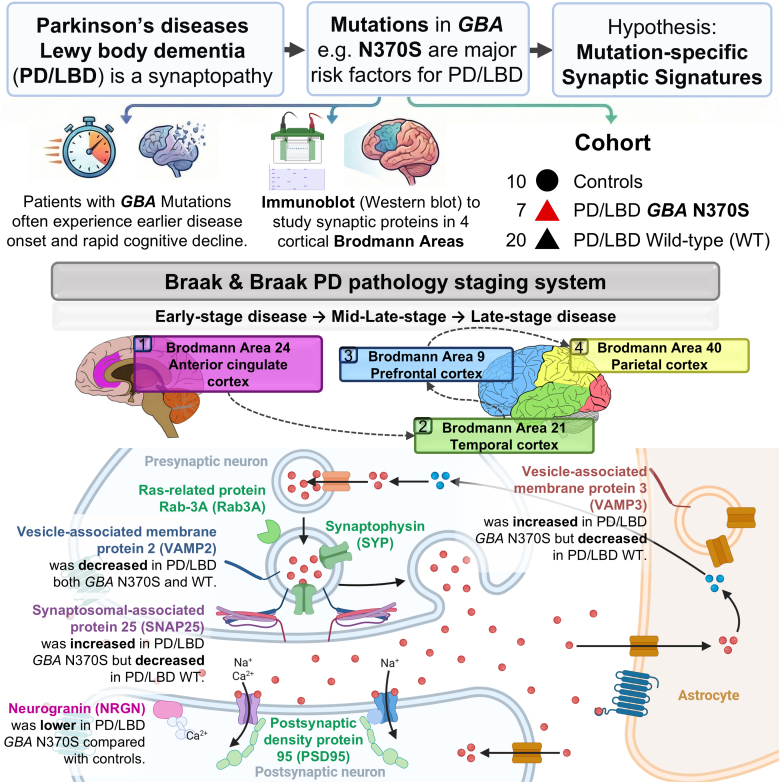

## Introduction

Glucocerebrosidase gene (*GBA*) mutations lead to deficient activity of the lysosomal enzyme, GCase, that causes Gaucher disease (GD). Furthermore, *GBA* mutations are a recognized risk factor for developing Parkinson’s disease (PD) and are also known to develop Lewy body dementia (LBD) which covers two closely related disorders namely dementia with Lewy bodies (DLB) and PD dementia (PDD). Clinically, PD patients with *GBA* mutations present with an early disease onset (early-mid fifties), six-fold increased cognitive decline, and rapid disease progression compared with non-carriers. AD, PDD, and DLB are the most common forms of neurodegenerative dementias.^1–3^ Cognitive impairment progressively declines in DLB and PDD patients, resulting in decreased quality of life, and increased mortality.^1,4,5^ Synapse loss and synaptic protein alterations have been proposed as important elements leading to dementia as they are concomitant with cognitive impairments from early phases of dementia.^6^ It has been shown that there is a better correlation between synaptic loss and cognitive deficit in AD than the hallmark tau and Aβ pathologies.^7–9^ Numerous studies have shown that changes in synaptic function are accompanied by changes in the concentration of synaptic proteins,^10^ and there is increasing attention on their role in synucleinopathies.^11–14^ Synaptopathies have been involved in a wide range of neurodegenerative diseases (AD, PD, PDD, DLB, and HD).^15,16^ Specific pre-synaptic proteins such as Rab3A, SNAP25, and SYP are crucial for vesicle trafficking, exocytosis and endocytosis, and have been found significantly altered in neurodegenerative diseases^14,17^ together with post-synaptic proteins such as NMDA and AMPA receptors, PSD95 and NRGN.^14,17–19^ Another study found an upregulation of post-synaptic protein PSD-95 in the dentate gyrus/CA3 regions in combination with downregulation of pre-synaptic proteins (synapsin1 and RIM3) in rat model.^20^ Furthermore, a recent study showed changes in the concentration of pre-synaptic proteins including SNAP25, and Rab3A along with a post-synaptic protein, NRGN, in post-mortem neocortical regions in PD, DLB and AD.^14^ A recent review^21^ has summarized alterations of pre-synaptic proteins not only in PD but also in DLB, PDD and rodent animal models. Some of these proteins include SYP, VAMP2, SNAP25 and PSD95.^21^

Herein we hypothesize that PD/LBD associated with *GBA* mutations may show abnormal synaptic changes compared with PD/LBD-WT which may predispose to dementia in LBD (PDD and DLB) by impairment of synaptic function. The aim of this study was to investigate the importance of synaptic changes in PD/LBD cohort associated with *GBA* mutation to provide a more detailed characterization and to find synaptic biomarkers that will assist in differential diagnosis from early stage of disease progression. Interestingly, we found four synaptic proteins namely NRGN, SNAP25, VAMP2 and VAMP3 were significantly altered among the three groups of controls, PD/LBD with mutated *GBA*, and PD-LBD-WT suggesting the importance of mutant *GBA* on synaptic proteins alterations and may be disease progression.

## Materials and methods

### Research cohorts

#### Case selection and the experimental design

The cases were obtained from the collection of the Queen Square Brain Bank (QSBB) at University College London Queen Square Institute of Neurology, which have been acquired for research using ethically approved protocols and are stored under a license from the Human Tissue Authority and request for tissue use is granted by local Tissue Request Committee (HTA license no: 12198; MTA reference No. QSBB13/16). Additional cases were requested from the South West Dementia Brain Bank (SWDBB) and added to the cohort. The research cohort for western blot analysis consisted of 3 groups; 10 controls, 7 PD/LBD-*GBA*, and 20 PD/LBD-WT that have been matched for disease duration and age of onset (Table 1). The demographic variables are summarized by cohort groups in Table 1 with full individual details provided in Supplementary Table 1.

**Table 1:** The demographic characteristics and the brain bank sources of each group in the cohort

Groups		Age (yrs)	Gender F/m	PMD (hrs)	pH	Age of onset (yrs)	Disease duration (yrs)	D/ND in LBD
**PD/LBD *GBA* N370S n** **=** **7**	**n**	7	7	7	5	7	7	7
	**mean**	80.78	0/7	51.71	6.26	67.14	13.57	3/4
	**SD**	7.84	-	31.59	0.09	8.43	7.41	-
**PD/LBD WT n** **=** **20**	**n**	20	20	20	6	20	20	20
	**mean**	83.54	4/16	60.35	6.03	68.80	14.73	11/9
	**SD**	5.26	-	32.45	0.15	10.46	6.89	-
**PD/LBD Total n** **=** **27**	**n**	27	27	27	11	27	27	7
	**mean**	82.83	4/23	58.11	6.14	68.37	14.43	14/13
	**SD**	6.00	-	31.85	0.17	9.84	6.90	-
**control n** **=** **10**	**n**	10	10	10	6	N/A	N/A	N/A
	**mean**	86.13	2/8	57.70	5.98	N/A	N/A	N/A
	**SD**	3.14	-	22.26	0.23	N/A	N/A	N/A
**Total n** **=** **37**	**n**	37	37	37	17	N/A	N/A	N/A
	**mean**	83.72	6/31	58	6.08	N/A	N/A	N/A
	**SD**	5.54	-	29.27	0.20	N/A	N/A	N/A

Brain bank	Control	PD/LBD *GBA* N370S	PD/LBD WT
**Queen Square Brain Bank (QSBB)**	8	5	17
**South West Dementia Brain Bank (SWDBB)**	2	2	3

n, the total number of cases used in this study; SD, standard deviation; the dash sign (-), not possible to calculate standard deviation; N/A, not applicable; PMD, Postmortem delay; pH, potential hydrogen levels of the tissue; D, have dementia; ND, no dementia; LBD, Lewy Body dementia; PDD, Parkinson’s disease with dementia + DLB, dementia with Lewy body; WT, Wild-Type.

Four cortical brain regions were included for this study according to the disease progression from early to late-stage disease: the anterior cingulate cortex [ACC; Brodmann area 24 (BA24)], temporal cortex [TC; Brodmann area 21 (BA21)], prefrontal cortex [PFC; Brodmann area 9 (BA9)], and parietal cortex [PC; Brodmann area 40 (BA40)]. ACC was selected for the early development of pathology encountered in this region in DLB and PDD,^22^ TC was selected for its association with cognitive decline as assessed by the MMSE in DLB cases,^13^ PFC was selected because of its proposed roles in executive function^23^ of which decline is a cardinal symptom of DLB and PDD, and PC was selected because of its pathologic predominance in AD as opposed to DLB and PDD^24–28^ as well as being the most late-stage development of pathology in PD/LBD patients. These four cortical brain regions show sequential distribution of α-synuclein from early to late-stage of the disease according to Braak & Braak staging system.^29^ To investigate synaptic dysfunction, neurons and astrocytes were obtained as majority of cell population by dissecting cortical grey matter and excluding white matter and meninges.

#### Selection of the seven synaptic markers

We focused our attention on four major markers representing pre-synaptic (SYP, and SNAP25), and post-synaptic (PSD-95 and NRGN) proteins that have been examined via standard immunoblot procedures in triplicate in four brain cortical regions (anterior cingulate, temporal, prefrontal, parietal) as previous findings by other groups have shown alteration in synaptic proteins in these cortical areas between several neurodegenerative diseases and controls.^9,21^ We also investigated additional set of pre-synaptic (Rab3A and VAMP2) markers that were found to be altered in the literature, and one astrocytic (VAMP3) protein to investigate the involvement of inflammatory pathway. These three markers were examined via standard immunoblot procedures and were limited to two brain regions (anterior cingulate and prefrontal cortices). These two brain regions were selected because they represented early, and late stage of the disease respectively. Although all the selected markers are mainly localized into the synapse, they all have different functions within synaptic boutons.^30,31^

#### Preparation of human brain tissue homogenate

The tissue was prepared for western blotting analysis as previously described.^24^ Tissue preparation for Western blotting was performed on a plate on dry ice and cortical grey matter was dissected from white matter and meninges. 150 mg of grey matter was homogenized in 3 mL on ice cold buffer containing 50 mM tris-HCL, 5 mM EGTA, 10mMEDTA, protease inhibitors (Sigma) and phosphatase inhibitors (Roche). Homogenization was performed using an IKA Ultra-Turrax mechanical probe (IKA Werke, Germany) for 15 s at high speed until the liquid was homogenous resulting in a crude homogenate. Finally, the crude homogenates were aliquoted into 3 separate Eppendorf tubes and immediately frozen on dry ice and stored at −70°C until processed for immunoblotting. This method was applied to each sample. Protein content was determined using Coomassie protein assay (Thermo Fisher #1856209) according to the manufacturer’s instructions.

#### Immunoblotting (western blotting)

Crude brain homogenate after protein determination was diluted with 5× sample buffer (Genscript MB1015), boiled for 5 min in a heat block at 95°C. Once cooled, the samples were stored at −20°C until required. 15-well 10% SDS-polyacrylamide gels were prepared to run samples. Samples were loaded at 20 µL (1 µg/µL) total protein on 10% sodium dodecyl sulphate-polyacrylamide gel (SDS-PAGE) electrophoresis for protein separation. TV200 twin-plate 20.5 × 10 cm (W × H) format mini-gel units were used (Scis-Plas, Cambridge, UK). On each gel the first lane was always 1.5 µL of full range rainbow molecular weight marker (MWM) (RPN800E, Sigma). Gels were run at 160 V for about 90 min. Afterward, gel contents were transferred onto nitrocellulose membranes (Hydro bond-C, Amersham) using electro blotting for mini gels (Scis-Plas, Cambridge, UK) in transfer buffer. All non-specific binding sites were blocked by incubating the membrane for 30 min at 25°C in 5% (w/v) dried skimmed milk (Marvel) dissolved in phosphate-buffered saline (PBS). After blocking, an appropriate primary antibody (Supplementary Table 2) was added and left on the rocker overnight in the cold room at 4°C (See table for dilutions and more details on primary antibody). After washing with phosphate-buffered saline and tween (PBST), the membranes were probed with the relevant secondary antibodies (Supplementary Table 2), either an IRDye 680LT goat anti-Mouse IgG (Licor Biosciences), visible under the red channel, or an IRDye 750CW goat anti-Rabbit, visible under the green channel (Licor Biosciences). Both secondary antibodies were added with dilutions of 1:5000 in 5% milk PBS for one hour at room temperature on the shaker. Finally, the membranes were washed three times for 5 min each with PBST.

#### Quantification of blots

The density of the lanes and bands was quantified using LI-COR^®^ Biosciences’ odyssey^®^ Infrared Imagine System, (software version 5.2). Rat cortex was used on each gel to normalize difference between each gel run. The relative protein quantification (RPQ) values were expressed as ratios to rat cortex and internal loading control β-tubulin.

#### Statistical analysis

Data analysis was carried out using GraphPad Prism 7 XML 7.03 (GraphPad, San Diego, California, USA). For each brain region (BA9, BA21, BA24 and BA40), expression levels of proteins between controls, PD/LBD-*GBA*, and PD/LBD-WT groups were compared statistically. To use parametric one-way ANOVA over non-parametric Kruskal–Wallis test (KW), dataset needs to be normally distributed. Our data were not normally distributed; therefore, differences in protein levels between groups were statistically analysed using the Kruskal–Wallis (KW) test, followed by the two-stage step-up Benjamini, Krieger, and Yekutieli (BKY-FDR) method as a post-hoc test to control the false discovery rate. The effect sizes *η*^2^_H_ was calculated by this formula (*H* − *k* + 1)/(*n* − *k*) where *H* is H-statistics of the KW tests, *k* is the number of groups, and *n* is the number of all samples combined.

Pearson and Spearman correlation analyses among region-matched pairs of synaptic proteins. Spearman correlations were conducted to analyse the trends of association between synaptic proteins and the 5 confounding variables (age, pH, PMD, age of onset, disease duration). Adjusted KW test was performed by first computing the residuals of the linear regression predicting a synaptic protein level from a confounding variable, then perform the KW test on the residuals across the groups.

Empirical power analysis was performed by resampling each group at varying equal sample size or the same sample size observed in the experiment for 500 rounds. In each round, Mann-Whitney tests were performed on each pair and *P*-values were recorded. Statistical power was then calculated as the ratio of *P*-values that are < 0.05. Then for each variable, Cohen’s *d* effect sizes were calculated given statistical power and corresponding sample sizes ranging from 6 to 20 and expressed as Median ± MAD.

## Results

### Four synaptic proteins in four cortical brain regions; (PSD-95, NRGN, SYP, SNAP25)/(anterior cingulate, temporal, prefrontal and parietal cortices)

The expression of the above mentioned four markers were examined in detail in four cortical brain regions (anterior cingulate, temporal, prefrontal, and parietal) representing early to late-stage disease, and between three groups (control, PD/LBD-*GBA*, and PD/LBD-WT).

#### Post-synaptic proteins; PSD-95 and NRGN

**Figure 1:**
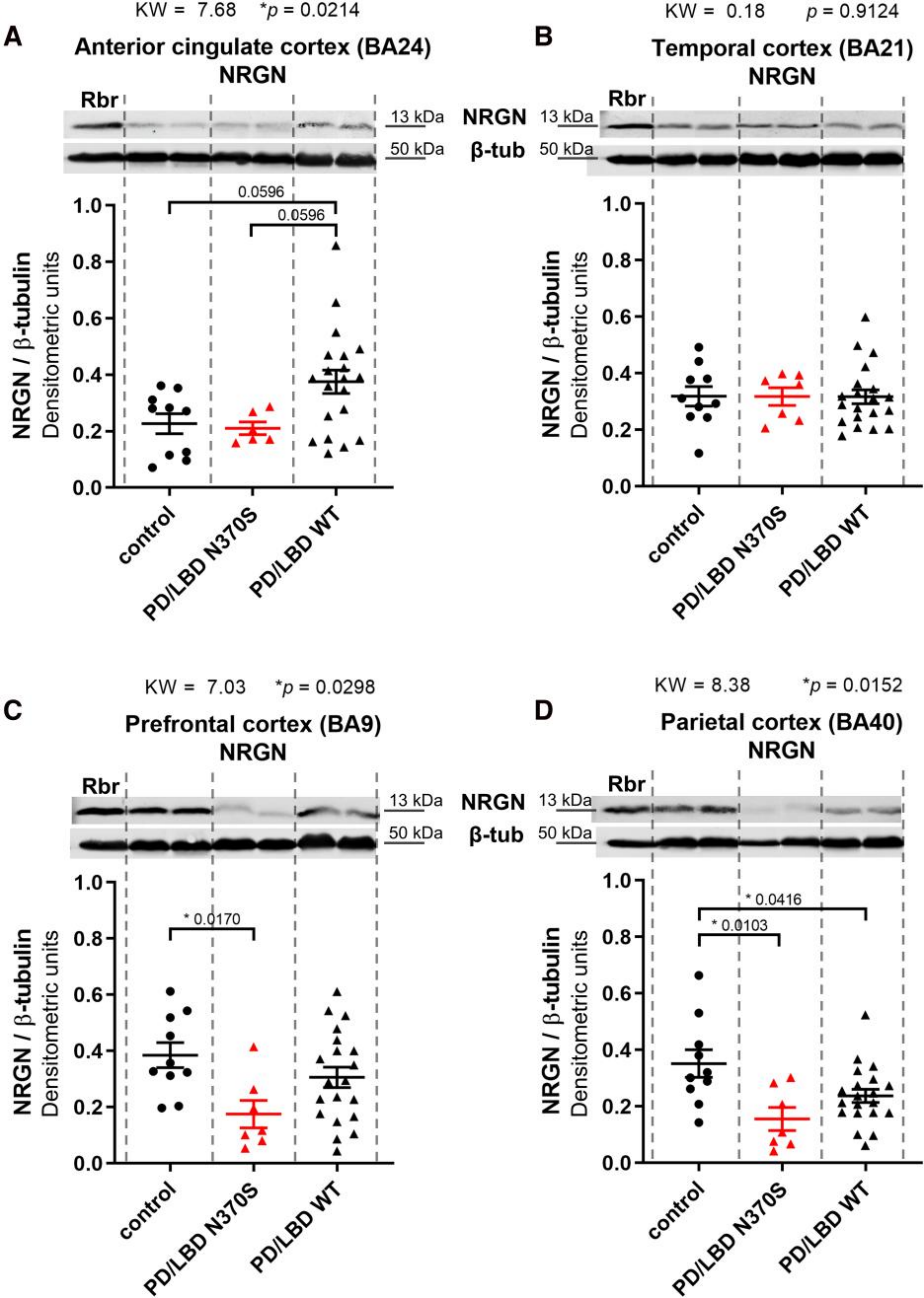
**Scatter plots of neurogranin (NRGN) normalized to β-tubulin across four cortical brain regions and three experimental groups. (A–D)** Neurogranin levels in the ACC (BA24), temporal cortex (BA21), prefrontal cortex (BA9), and parietal cortex (BA40), respectively. Kruskal-Wallis (KW) tests were performed for each brain region, yielding effect sizes (*η*^2^H) of 0.172, 0.000, 0.148 and 0.187, respectively. Black dots signify cognitively healthy controls, while red triangles and black triangles signify PD/LBD patients with the *GBA1* N370S mutation and *GBA1* WT, respectively. Bars represent mean ± standard error of the mean (SEM). Exact *P*-values are displayed for selected post-hoc comparisons (*P < 0.05). The cropped blots displayed are examples of representative Western blots (WB); full-length images of the WB membranes are available in the Supplementary Materials. The corresponding positional codes for the representative bands on the full membranes (where letters indicate the membrane/row ID and numbers indicate the column/lane ID) are as follows: (**A**) = Supplementary Fig. 8 [AR, D5, D6, A2, A6, C2, C7]; (**B**) = Supplementary Fig. 9 [AR, E1, E3, A5, A6, B1, B2]; (**C**) = Supplementary Fig. 10 [BR, D6, D7, A5, A6, B2, B3]; (**D**) = Supplementary Fig. 11 [DR, E1, E2, A3, A6, D2, D4]. Rbr denotes the first lane loaded with rat brain extract as an internal control across gels; BA, Brodmann Area; kDa, kilodalton.

Immunoblot analysis of PSD-95 and its semiquantitation over β-tubulin levels did not show any change between the two disease sub-groups or controls (Supplementary Fig. 1, with the corresponding full membrane images shown in the Supplementary Figs 4–7). The semi-quantification data of NRGN expression levels over β-tubulin were analysed, are presented in Fig. 1 (the corresponding full membrane images shown in the Supplementary Figs 8–11) from four brain regions between three groups with *P*-values from KW test. Although the NRGN levels in anterior cingulate cortex showed a significant total *P*-value (KW = 7.68; *P* = 0.0214), the pairwise test showed that NRGN levels were increased in PD/LBD-WT group compared with controls and mutant *GBA* groups (*P* = 0.0596, for both), although this did not reach significance. In the temporal cortex, there was no difference in NRGN levels between any groups. In the prefrontal cortex (KW = 7.03; *P* = 0.0298), NRGN was significantly reduced in PD/LBD-*GBA* group compared with controls (*P* = 0.0170). In the parietal cortex (KW = 8.38; *P* = 0.0152), NRGN was significantly reduced in both diseased groups (*GBA P* = 0.0103, WT *P* = 0.0416) compared with controls.

#### Pre-synaptic proteins; SYP and SNAP25

**Figure 2:**
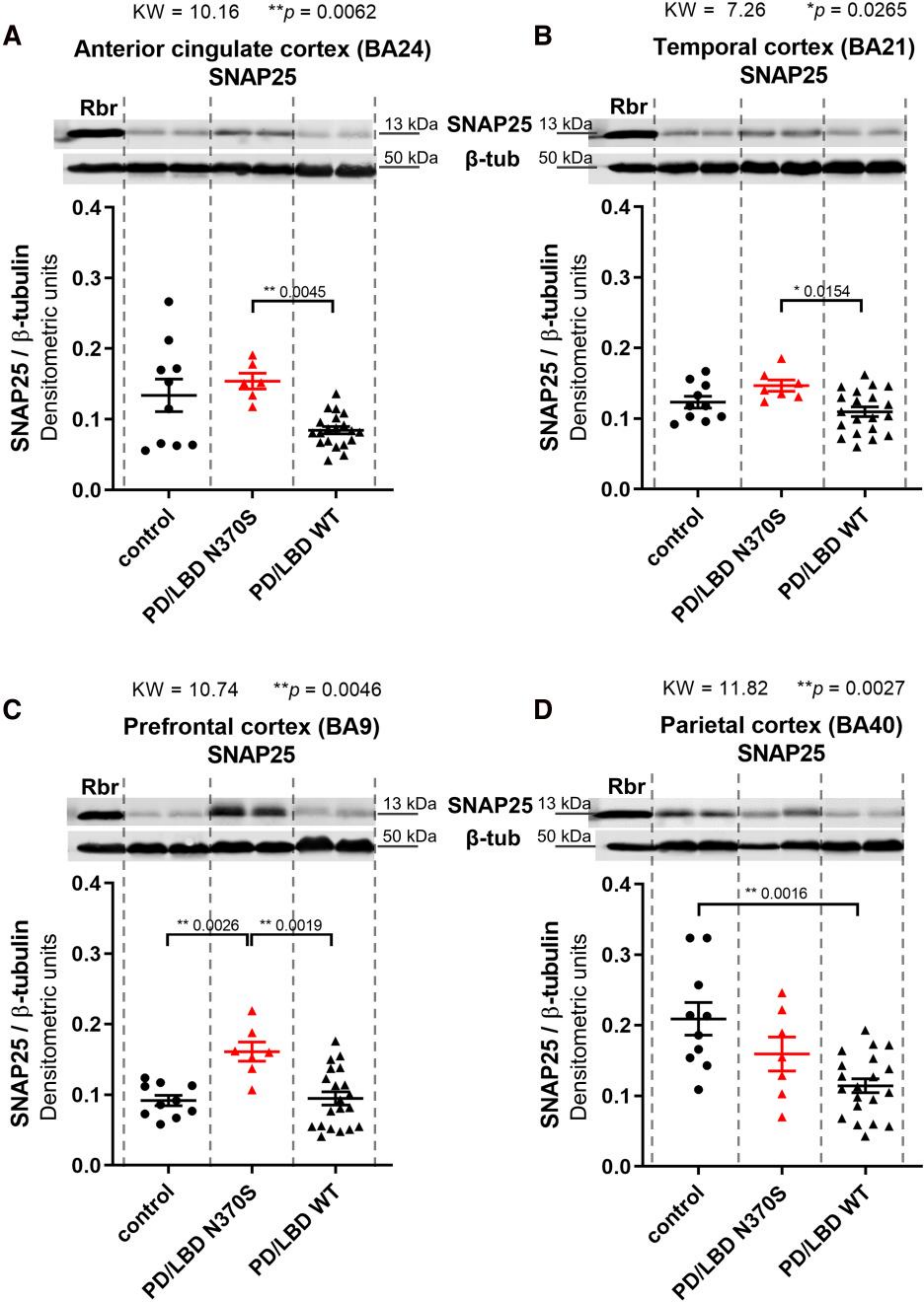
**Scatter plots of SNAP25 normalized to β-tubulin across four cortical brain regions and three experimental groups. (A–D)** SNAP25 levels in the ACC (BA24), temporal cortex (BA21), prefrontal cortex (BA9), and parietal cortex (BA40), respectively. Kruskal-Wallis (KW) tests were performed for each brain region, yielding effect sizes (*η*^2^H) of 0.247, 0.155, 0.258 and 0.289, respectively. Black dots signify cognitively healthy controls, while red triangles and black triangles signify PD/LBD patients with the *GBA1* N370S mutation and *GBA1* WT, respectively. Bars represent mean ± standard error of the mean (SEM). Exact *P*-values are displayed for selected post-hoc comparisons (*P < 0.05, **P < 0.01). The cropped blots displayed are examples of representative Western blots (WB); full-length images of the WB membranes are available in the Supplementary Materials. The corresponding positional codes for the representative bands on the full membranes (where letters indicate the membrane/row ID and numbers indicate the column/lane ID) are as follows: (**A**) = Supplementary Fig. 8 [AR, D5, D6, A2, A6, C2, C7]; (**B**) = Supplementary Fig. 9 [AR, E1, E3, A5, A6, B1, B2]; (**C**) = Supplementary Fig. 10 [BR, D6, D7, A5, A6, B2, B3]; (**D**) = Supplementary Fig. 11 [DR, E1, E2, A3, A6, D2, D4]. Rbr denotes the first lane loaded with rat brain extract as an internal control across gels; BA, Brodmann Area; kDa, kilodalton.

Densitometric analysis of SYP over β-tubulin levels ratio has been displayed in scatter plots from four cortical brain regions between three groups with *P*-values from KW test (Supplementary Fig. 2, with the corresponding full membrane images shown in the Supplementary Figs 4–7). In cingulate cortex (KW = 6.281; *P* = 0.0433), although the total *P*-value was significant, SYP was non-significantly increased in PD/LBD-WT group compared with controls (*P* = 0.0647). In the remaining cortical brain regions (temporal. prefrontal, and parietal), there was no change between disease or control groups. Additionally, the semi-quantification data of SNAP25 expression levels over β-tubulin were analysed similarly, and are presented in scatter plots from four brain regions between three groups with the *P*-values from KW test (Fig. 2, with the corresponding full membrane images shown in the Supplementary Figs 8–11). In anterior cingulate cortex (KW = 10.16; *P* = 0.0062), SNAP25 was significantly reduced in wild type group compared with mutant *GBA* group (*P* = 0.0045). In temporal cortex (KW = 7.26; *P* = 0.0265), SNAP25 was significantly increased in the mutant *GBA* group compared with wild type group (*P* = 0.0154). In prefrontal cortex (KW = 10.74; *P* = 0.0046), SNAP25 was significantly increased in mutant *GBA* group compared with control and wild type groups (*P* = 0.0026, *P* = 0.0019, respectively). In parietal cortex (KW = 11.82; *P* = 0.0027), SNAP25 was significantly reduced in wild type group compared with controls (*P* = 0.0016).

### Analysis of pre-synaptic protein; Rab3A and VAMP2, and astrocytic protein; VAMP3 in two brain regions (prefrontal and cingulate)

Two additional synaptic markers (Rab3A, VAMP2) were investigated in prefrontal and cingulate cortices. A third marker (VAMP3) was selected to investigate the involvement of the astrocytes in these regions. The expression of the above three markers were examined in two cortical brain regions (anterior cingulate, prefrontal) because these two regions showed the most significant changes in synaptic proteins as displayed in previous analysis (Figs 1, 2A, and C) of the four pre-synaptic, and post-synaptic proteins, and simultaneously they represent early, and late-stage of the disease, respectively. These three markers were found significantly altered across different synucleinopathies in the literature, therefore, they were investigated in the three groups (control, PD/LBD-*GBA* and PD/LBD-WT) to identify whether the presence of a *GBA* mutation has a role on the expression level of these markers.

**Figure 3:**
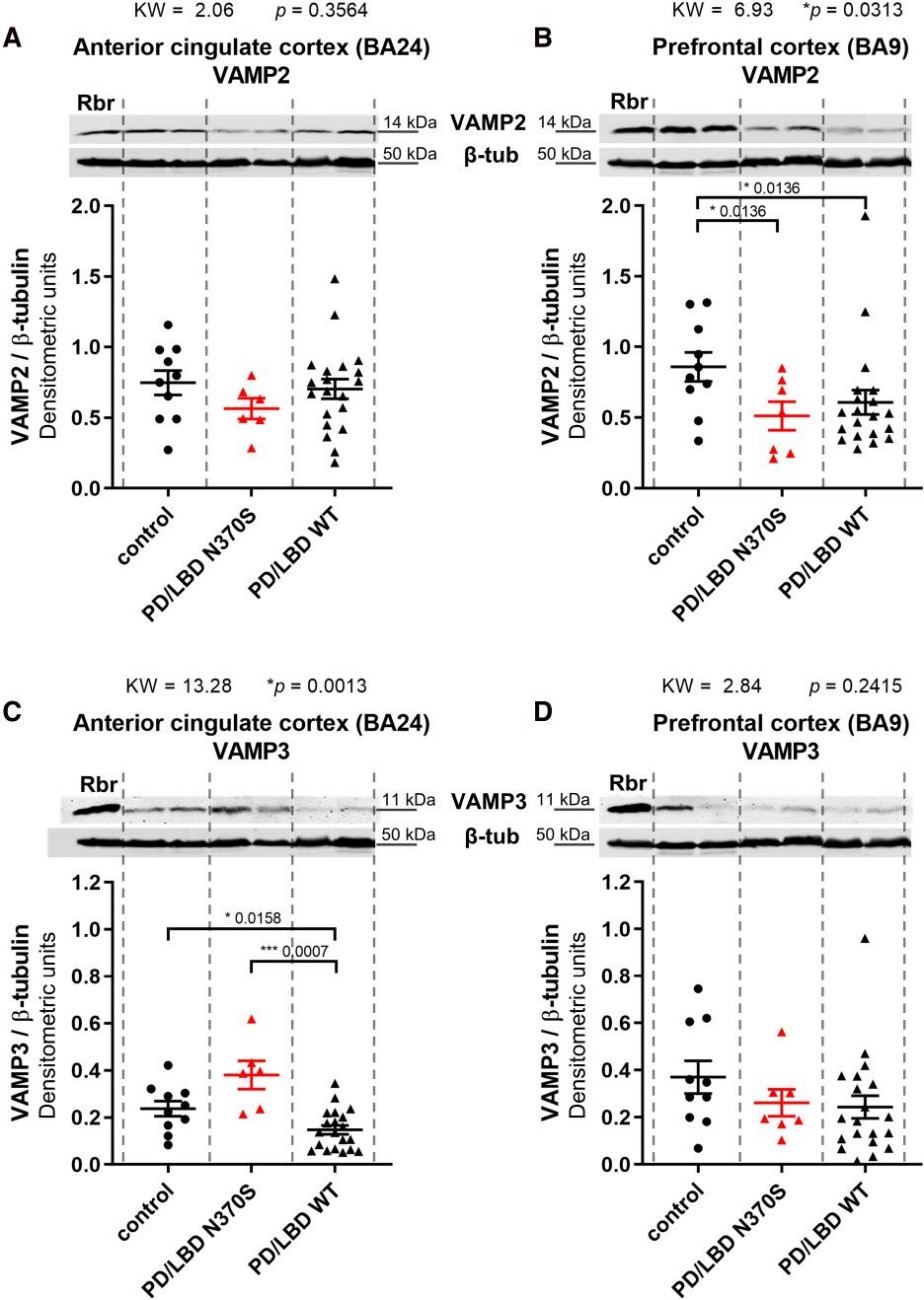
**Scatter plots of VAMP2 and VAMP3 normalized to β-tubulin across two cortical brain regions and three experimental groups.** (**A**, **B**) VAMP2 and (**C**, **D**) VAMP3 levels in the ACC (BA24) and prefrontal cortex (BA9), respectively. Kruskal-Wallis (KW) tests were performed for each brain region, yielding effect sizes (*η*^2^H) of 0.002, 0.145, 0.342 and 0.025 respectively. Black dots signify cognitively healthy controls, while red triangles and black triangles signify PD/LBD patients with the *GBA1* N370S mutation and *GBA1* WT, respectively. Bars represent mean ± standard error of the mean (SEM). Exact *P*-values are displayed for selected post-hoc comparisons (*P < 0.05, **P < 0.01, ***P < 0.001). The cropped blots displayed are examples of representative Western blots (WB); full-length images of the WB membranes are available in the Supplementary Materials. The corresponding positional codes for the representative bands on the full membranes (where letters indicate the membrane/row ID and numbers indicate the column/lane ID) are as follows: (**A**) = Supplementary Fig. 12 [ER, E1, E2, A1, A4, C5, D2]; (**B**) = Supplementary Fig. 13 [CR, E3, E4, A3, A7, B5, C6], (**C**) = Supplementary Fig. 12 [ER, E1, E2, A1, A4, C5, D2]; (**D**) = Supplementary Fig. 13 [CR, E3, E4, A3, A7, B5, C6]. Rbr denotes the first lane loaded with rat brain extract as an internal control across gels; BA, Brodmann Area; kDa, kilodalton.

Semi-quantification data of Rab3A expression levels over β-tubulin showed no difference between controls, and any of the diseased groups in either cortical brain regions (anterior cingulate, and prefrontal) (Supplementary Fig. 3, with the corresponding full membrane images shown in the Supplementary Figs 12 and 13). Western blot analysis of VAMP2 over β-tubulin ratio has been presented in a scatter plot from two cortical brain regions between three groups with *P*-values from KW test (Fig. 3, with the corresponding full membrane images shown in the Supplementary Figs 12 and 13). In anterior cingulate cortex, there was no change between the controls and any of the diseased groups. In prefrontal cortex (KW = 6.93; *P* = 0.0313), VAMP2 was significantly reduced in both diseased groups (*P* = 0.0136, for both) compared with controls.

Immunoblot analysis of VAMP3 and its semi-quantitation over β-tubulin levels is displayed in scatter plot in two brain regions between three groups with *P*-values from KW test (Fig. 3). In the anterior cingulate cortex (KW = 13.28; *P* = 0.0013), VAMP3 was significantly reduced in PD/LBD-WT group compared with controls and mutant *GBA* groups (*P* = 0.0158, *P* = 0.0007, respectively). In the prefrontal cortex, there was no significant change between groups, although both diseased groups displayed a reduction compared with controls.

### Correlation and adjustment for confounding variables

Non-parametric rank-based Spearman correlations were computed between region-matched synaptic protein variables (Table 2). SYP was significantly correlated with the postsynaptic PSD95 in all regions [(BA24, rs = 0.4541), (BA21, rs = 0.5465), (BA40, rs = 0.4481)], except BA9. SYP was also significantly correlated with VAMP2 in both BA9 and BA24. NRGN and SNAP25 tend to be negatively correlated. Surprisingly, SNAP25 is also negatively correlated with other presynaptic proteins SYP and VAMP2. VAMP2 and Rab3A, besides highly correlated with one another in both regions [(BA24, rs = 0.5653), (BA9, rs = 0.5446)], also showed similar patterns of correlation with PSD95 only in BA24 and significant correlation with SYP but not SNAP25. Rab3A is also positively correlated with the postsynaptic NRGN in the BA24. VAMP3, on the other hand, was only correlated with SNAP25 in BA24 and VAMP2 in BA9. Aside from VAMP3, SNAP25 was negatively correlated with other proteins such as SYP and NRGN in BA24.

**Table 2:** Region-matched Spearman correlation coefficients among synaptic proteins

Variable 1	Variable 2	Spearman *rs*	Variable 1	Variable 2	Spearman *rs*
SYP_BA24	PSD95_BA24	**0**.**4541**	SYP_BA9	NRGN_BA9	**0**.**5180**
SYP_BA21	PSD95_BA21	**0**.**5465**	SYP_BA21	NRGN_BA21	−0.1081
SYP_BA9	PSD95_BA9	0.1866	SYP_BA24	NRGN_BA24	0.3107
SYP_BA40	PSD95_BA40	**0**.**4481**	SYP_BA40	NRGN_BA40	−0.2307
SNAP25_BA9	PSD95_BA9	−0.0965	SNAP25_BA24	NRGN_BA24	**−0**.**3272**
SNAP25_BA21	PSD95_BA21	−0.0581	SNAP25_BA21	NRGN_BA21	−0.1048
SNAP25_BA24	PSD95_BA24	−0.1977	SNAP25_BA9	NRGN_BA9	**−0**.**4021**
SNAP25_BA40	PSD95_BA40	**−0**.**3450**	SNAP25_BA40	NRGN_BA40	0.1019
SYP_BA24	SNAP25_BA24	**−0**.**4461**	PSD95_BA24	NRGN_BA24	0.2345
SYP_BA21	SNAP25_BA21	0.1458	PSD95_BA21	NRGN_BA21	−0.0040
SYP_BA9	SNAP25_BA9	−0.1970	PSD95_BA9	NRGN_BA9	**0**.**3291**
SYP_BA40	SNAP25_BA40	0.0500	PSD95_BA40	NRGN_BA40	−0.0503
SYP_BA24	Rab3A_BA24	**0**.**5882**	PSD95_BA24	Rab3A_BA24	**0**.**3318**
SYP_BA9	Rab3A_BA9	**0**.**4651**	PSD95_BA9	Rab3A_BA9	0.0548
SNAP25_BA24	Rab3A_BA24	−0.0793	NRGN_BA24	Rab3A_BA24	**0**.**4111**
SNAP25_BA9	Rab3A_BA9	0.2760	NRGN_BA9	Rab3A_BA9	0.0955
SYP_BA24	VAMP2_BA24	**0**.**5277**	PSD95_BA24	VAMP2_BA24	**0**.**3328**
SYP_BA9	VAMP2_BA9	**0**.**4986**	PSD95_BA9	VAMP2_BA9	0.0635
SNAP25_BA24	VAMP2_BA24	−0.1369	NRGN_BA24	VAMP2_BA24	0.1709
SNAP25_BA9	VAMP2_BA9	−0.1064	NRGN_BA9	VAMP2_BA9	0.2921
Rab3A_BA24	VAMP2_BA24	**0**.**5653**	Rab3A_BA24	VAMP3_BA24	−0.1725
Rab3A_BA9	VAMP2_BA9	**0**.**5446**	Rab3A_BA9	VAMP3_BA9	0.1937
SYP_BA24	VAMP3_BA24	−0.1153	PSD95_BA24	VAMP3_BA24	−0.0517
SYP_BA9	VAMP3_BA9	−0.1626	PSD95_BA9	VAMP3_BA9	−0.0038
SNAP25_BA24	VAMP3_BA24	**0**.**4782**	NRGN_BA24	VAMP3_BA24	−0.1810
SNAP25_BA9	VAMP3_BA9	0.0903	NRGN_BA9	VAMP3_BA9	−0.0443
VAMP2_BA24	VAMP3_BA24	0.1143			
VAMP2_BA9	VAMP3_BA9	**0**.**4879**			

Bolded = Statistically significant correlation coefficients.

To investigate the potential effects of confounding variables, Spearman correlations were first conducted between any given synaptic proteins and one of demographic variables including pH, PMD, age at death, age of onset, and disease duration (Table 3). Unlike other variables, the number of samples with pH data available is only 17. While pH is negatively correlated with NRGN in BA9 (*r* = −0.5515) and BA40 (*r* = −0.6985), pH is positively correlated with SNAP25 in BA24 (*r* = 0.7676) and BA21 (*r* = 0.5025). PMD is expectedly negatively correlated with most proteins especially PSD95 [(BA24, *r* = −0.2833), (BA21, *r* = −0.3936), (BA9, *r* = −0.4473), (BA40, *r* = −0.5004)] and VAMP2 [(BA24, *r* = −0.3517), (BA9, *r* = −0.1923)]. While Age at death and age of onset were not significantly correlated with any proteins, the age of onset and disease duration expectedly show trends of opposite directions such as for NGRN and PSD95.

**Table 3:** Spearman correlations between synaptic proteins of different regions (rows) and demographic variables (columns)

	Demographic				
Synaptic	pH	PMD	Age_Death	Age_Onset	Duration
PSD95_BA24	−0.1206	−0.2833	−0.0496	0.1894	−0.0657
PSD95_BA21^a^	−0.2279	**−0**.**3936**	0.1764	0.2714	−0.3415
PSD95_BA9^a^	−0.3064	**−0**.**4473**	0.1139	0.1544	−0.1938
PSD95_BA40^a^	−0.2353	**−0**.**5004**	0.0530	0.1993	**−0**.**3916**
NRGN_BA24^a^	*−0*.*3294*	−0.0805	−0.2907	−0.1616	0.0438
NRGN_BA21^a^	−0.1348	**−0**.**3234**	0.1002	−0.1645	**0**.**4106**
NRGN_BA9^a^	**−0**.**5515**	−0.1275	0.3071	0.0685	0.1345
NRGN_BA40^a^	**−0**.**6985**	−0.0593	0.1018	−0.2485	**0**.**3898**
SYP_BA24	0.0235	0.1349	0.0432	0.0459	0.0794
SYP_BA21	−0.2279	−0.1324	0.2037	0.2186	−0.2299
SYP_BA9^a^	*−0*.*4534*	0.0157	0.1695	0.1718	−0.0697
SYP_BA40	0.1863	−0.1848	−0.0608	−0.2109	0.0642
SNAP25_BA24^a^	**0**.**7676**	−0.1236	−0.0085	−0.1852	0.1777
SNAP25_BA21^a^	**0**.**5025**	0.2343	−0.0080	−0.0177	0.0388
SNAP25_BA9	0.2010	−0.1170	−0.2610	−0.2005	0.1042
SNAP25_BA40	0.2034	0.0965	0.0882	−0.2274	0.3302
Rab3A_BA24	0.1324	−0.1070	0.0107	0.0746	0.0267
Rab3A_BA9	0.0564	0.0234	−0.1570	−0.1214	0.1186
VAMP2_BA24^a^	−0.2765	**−0**.**3517**	0.2703	0.0894	0.0329
VAMP2_BA9	−0.0564	−0.1923	0.2374	0.0446	0.0758
VAMP3_BA24^a^	**0**.**5324**	−0.2209	0.0395	−0.1171	0.1555
VAMP3_BA9	0.1176	−0.2760	0.0230	−0.1758	0.1024

Bolded = Statistically significant correlation coefficients.

*Italicized* = Strong correlation coefficients with pH but not statistically significant because the sample size of valid pH data (*N* = 17) is lower than other demographic variable (*N* = 37).

^a^Synaptic proteins with at least one strong correlation (bolded or italicized coefficient).

To explore the potential effects of these confounding variables, statistical tests were performed after rank-based adjustment for respective demographic variables as follows: bootstrapped KW test for pH; raw KW tests for PMD and age at death; and Mann-Whitney U-test for age of onset and disease duration in PD/LBD *GBA1* N370S versus WT (Table 4). The data for adjustment by pH had to be bootstrapped or resampled with replacement for equal sample size (control *N* = 10, PD/LBD N370S *N* = 7, PD/LBD WT *N* = 20) given limited number of samples with available pH (control *N* = 6, PD/LBD N370S *N* = 5, PD/LBD WT *N* = 6). The bootstrapping was repeated for 200 times to give the mean unadjusted KW and pH-adjusted KW *P*-values. The R-squared of mean bootstrapped unadjusted KW *P*-values with respect to the original *P*-values was sufficiently high at 0.8658, validating their potentials to represent the original raw data. In concordance with the spearman correlations in Table 3, adjusting for pH increased *P*-values for NRGN (BA24, BA9, BA40) and SNAP25 BA24, which showed large magnitude of correlation with the pH. This result was also in line with the finding that pH of the PD/LBD N370S group was significantly higher than that of control (*P* = 0.0303; 6.264 versus 5.977 respectively). For the KW tests on raw data, NRGN and SYP showed slight increase in *P*-value with loss of significance after adjusting for PMD and age at death [(NRGN_BA24: 0.0214 → 0.0511, 0.0733), (SYP_BA24: 0.0460 → 0.0767, 0.0767)]. Adjustment for age at death also increased *P*-values in NRGN and VAMP2 in the BA9. Since age of onset and disease duration was not present in the control group, the effects of these two variables can only be accounted for when comparing the two disease groups: PD/LBD *GBA1* N370S versus WT. The most pronounced impact of adjustment by age of onset or disease duration was observed in the NRGN of BA24 where the *P*-value was increased from 0.0596 to 0.2207 and 0.2674 respectively.

**Table 4:** KW test *P*-values after rank-based adjustment by demographic variables

	Bootstrapped KW test: *P*-value		Raw KW test: *P*-value			MWU—PD/LBD (N370S versus WT): *P*-value		
Synaptic	base	by pH	base	by PMD	by Age_Death	base	by Age_Onset	by Duration
PSD95_BA24	0.2771	0.1037	0.2518	0.1572	0.2700	0.8823	0.4640	0.4980
PSD95_BA21	0.4008	0.4341	0.8660	0.8083	0.9353	0.8926	0.8926	0.8926
PSD95_BA9	0.4053	0.4271	0.8901	0.7517	0.7947	0.8926	0.7662	0.8500
PSD95_BA40	0.3541	0.3372	0.9254	0.7803	0.9056	0.9354	0.9354	0.8926
NRGN_BA24	**0**.**0478**	^a^0.0903	**0**.**0214**	^a^0.0511	^a^0.0733	0.0596	0.2207	0.2674
NRGN_BA21	0.4564	0.4136	0.9124	0.8255	0.8878	0.7253	0.8079	0.6071
NRGN_BA9	**0**.**0294**	^a^0.2605	**0**.**0298**	**0**.**0250**	^a^0.1043	0.0823	0.0631	0.1192
NRGN_BA40	**0**.**0045**	^a^0.1211	**0**.**0152**	**0**.**0145**	**0**.**0260**	0.1618	0.1455	0.1455
SYP_BA24	0.0558	0.0596	**0**.**0460**	^a^0.0767	^a^0.0767	0.1229	0.3998	0.3998
SYP_BA21	0.3921	0.4173	0.3570	0.3053	0.3386	0.8500	0.7253	0.8079
SYP_BA9	0.1264	0.1873	0.1217	0.1243	0.1522	0.6456	0.6456	0.6071
SYP_BA40	**0**.**0324**	**0**.**0264**	0.1114	0.1407	0.1309	0.0722	0.0823	0.0722
SNAP25_BA24	**0**.**0055**	**0**.**0501**	**0**.**0062**	**0**.**0025**	**0**.**0025**	**0**.**0045**	**0**.**0012**	**0**.**0012**
SNAP25_BA21	0.1006	0.1802	**0**.**0265**	**0**.**0148**	**0**.**0265**	**0**.**0154**	**0**.**0154**	**0**.**0154**
SNAP25_BA9	**0**.**0027**	**0**.**0030**	**0**.**0046**	**0**.**0043**	**0**.**0120**	**0**.**0019**	**0**.**0048**	**0**.**0015**
SNAP25_BA40	**0**.**0014**	**0**.**0014**	**0**.**0027**	**0**.**0026**	**0**.**0035**	0.1057	0.0935	0.0823
Rab3A_BA24	0.2284	0.2830	0.1661	0.1468	0.1793	0.4570	0.8926	0.8926
Rab3A_BA9	0.2361	0.2355	0.1968	0.1968	0.2298	0.0935	0.1057	0.0722
VAMP2_BA24	0.2681	0.4024	0.3564	0.5780	0.9016	0.2681	0.6456	0.6456
VAMP2_BA9	0.0741	0.0749	**0**.**0313**	**0**.**0408**	^a^0.0663	0.6850	0.6850	0.6850
VAMP3_BA24	**0**.**0042**	**0**.**0088**	**0**.**0013**	**0**.**0010**	**0**.**0005**	**0**.**0007**	**0**.**0001**	**0**.**0002**
VAMP3_BA9	0.2128	0.1265	0.2415	0.1883	0.2415	0.6456	0.7253	0.6456

Bolded = Statistically significant KW *P*-values.

^a^Adjusted *P*-values that become non-significant compared with their corresponding base original unadjusted *P*-values.

Empirical power analysis was conducted to study the relationship of increasing sample sizes on statistical power for the most potential pair-wise difference and estimate the empirical Cohen’s D effect size for each variable (Supplementary Fig. 14). As expected, the pairs that showed significant differences in the immunoblot has higher statistical power than others given the same experimental sample sizes but with few of them slightly lower than 80% namely NRGN BA24 (0.748) and VAMP2 BA9 (0.686). Notably, this analysis showed that significant difference in SYP across groups including PD/LBD N370S versus wildtype may manifest if sample sizes are increased and that BA21 in general is more robust to PD/LBD-associated synaptopathies.

## Discussion

### Summary of the key altered synaptic markers

Seven markers including pre-synaptic, post-synaptic, and astrocytic proteins have been investigated by WB, and several key findings were established in this investigation. The first set of four synaptic markers were examined in four cortical brain regions (anterior cingulate, temporal, prefrontal, and parietal cortices), of which lesions correspond to early, and late-stage disease according to Braak and Braak staging system.^29^ Among the four markers only NRGN, SYP and SNAP25 found to be significantly and regionally altered between groups. The expression of the post-synaptic protein, NRGN, was always the lowest in the PD/LBD-*GBA* group, and in three cortical brain regions (anterior cingulate, prefrontal and parietal), but only significantly lower than control in prefrontal, and parietal cortices. The pre-synaptic protein SYP was significantly increased in PD/LBD-WT group compared with the controls as per the overall *P*-value, but the post-hoc pairwise test does not show significance. The other pre-synaptic marker, SNAP25, was significantly increased in PD/LBD-*GBA* group compared with PD/LBD-WT group in three cortical brain regions (anterior cingulate, temporal, and prefrontal), but a significant increase compared with controls was only seen in prefrontal cortex. Furthermore, SNAP25 was always significantly decreased in PD/LBD-WT group in all four cortical brain regions examined; however, a significant decrease compared with controls was only seen in parietal cortex.

The second set of additional synaptic markers were examined and limited to anterior cingulate and prefrontal cortices, of which lesions correspond to early, and late-stage disease respectively, and they represent the most significant change brain areas among the four cortical brain regions selected in the first set of synaptic markers. Only the vesicular proteins VAMP2 and VAMP3 found to be regionally, and significantly altered. The pre-synaptic protein VAMP2 was reduced in both diseased groups compared with controls in both cortical brain regions (anterior cingulate, and prefrontal), Interestingly, a significant reduction of VAMP2 was observed in the late-stage disease affected area, the prefrontal cortex. The astrocytic protein VAMP3 was increased in PD/LBD-*GBA* cases but was simultaneously reduced in PD/LBD-WT groups compared with controls in cingulate cortex.

### Four proteins in four cortical brain regions; (PSD-95, neurogranin, synaptophysin, SNAP25)/(prefrontal, temporal, cingulate and parietal cortices)

#### PSD-95, and NRGN (post-synaptic proteins)

PSD-95 is the most abundant scaffolding protein in the excitatory postsynaptic density (PSD).^32^ PSD-95 level was not altered in any of the four cortical brain regions, and between any of the three studied groups. This could be due to multiple factors such as small sample size and/or different methodological approach between this study and other studies. The fact that the mutant *GBA* group behaved similarly to WT group, suggesting that this marker may share common mechanisms in PD pathogenesis.

The other post-synaptic marker, NRGN, is expressed mostly in the brain, specifically in dendritic spines.^33^ NRGN was significantly reduced in the mutant *GBA* groups compared with controls in prefrontal and parietal cortices, representing the late-stages of the disease. In fact, NRGN levels were consistently the lowest in PD/LBD-*GBA* group in three cortical brain regions (anterior cingulate, prefrontal and parietal). This phenomenon of NRGN alteration in PD/LBD-*GBA* group was not seen with either PSD-95 or SYP proteins, suggesting that *GBA* may alter the pathway of specific synaptic proteins, and this could be one of the reasons why patients with *GBA* mutations develop dementia earlier in disease. In fact, cerebrospinal fluid (CSF) NRGN are used to predict future cognitive decline in cognitively normal patients,^34^ and this post-synaptic membrane protein can be a useful marker for early diagnosis of cognitive decline. Furthermore, the present data strongly suggest that NRGN plays an important role in synaptic plasticity, synaptic regeneration, and long-term potentiation (LTP) mediated by the calcium- and calmodulin-signalling pathways.^35^ Disrupted synaptic structure reduce NRGN binding to its partner, calmodulin. This process affects the transmission of Ca^2+^ between the synapses and the formation of LTP, resulting in early cognitive decline.^36,37^ Therefore, NRGN reduction might alter synaptic function.

Studies in humans have shown that NRGN expression was decreased in AD patients particularly in the frontal and parietal cortices.^37,38^ Interestingly, in our study NRGN levels were significantly reduced in both diseased groups (with and without *GBA* mutation) compared with controls in the same brain regions which represent mid-late, and late-stage of PD Braak staging respectively. This reduction suggests that these regions are the most affected areas, and the region-specific similarity of change between the two diseases also could suggest a shared pathway for NRGN in AD, and PD. Furthermore, the similar pattern of change in prefrontal, and parietal cortices in both diseased groups compared with controls could also suggest a shared molecular pathway for NRGN in PD/LBD-WT and PD/LBD-*GBA* groups. However, since the disease is in its more aggressive form in the *GBA* carriers than in the WT group, NRGN levels were always much more reduced in the PD/LBD-*GBA* group despite the small size number compared with PD/LBD-WT or control groups in three cortical brain regions (anterior cingulate, prefrontal and parietal). This suggests that *GBA* mutation may play a role in increasing cognitive decline in the *GBA* carriers via altering the levels of specific synaptic markers such as NRGN. This could explain why *GBA* carriers develop cognitive decline at earlier age compared with non-carriers.

Our study showed bands of higher molecular weights species (HMW) than the expected size of 13 kD. Furthermore, these species appear under fully reducing conditions (DTT), which distinguishes them from previously reported disulphide-linked NRGN oligomers that are sensitive to reduction.^39^ We propose that these DTT-resistant bands represent bona fide HMW species of NRGN arising from several non-mutually exclusive mechanisms, which we have now clarified in the manuscript.

First, NRGN may form exceptionally stable, non-covalent oligomers that are resistant to denaturation by SDS. This characteristics of SDS-stable oligomer is well-documented for other intrinsically disordered proteins including those involved in neurodegeneration i.e. α-synuclein, amyloid-β, and tau^40–42^ and even those crucial in plant freezing tolerance.^43^ Supporting this, a commercial datasheet for recombinant NRGN explicitly lists ‘dimerization, multimerization’ as a potential cause for anomalous migration even under reducing conditions.^44^ The bands we observe at ∼30 kDa and ∼45 kDa are consistent with the migration of such stable dimers and trimers. Second, these bands could represent post-translationally modified forms of NRGN. The covalent attachment of a single Small Ubiquitin-like Modifier (SUMO; ∼11 kDa) or a ubiquitin moiety (∼8.5 kDa) would generate species with apparent molecular weights consistent with our observations (∼26 kDa and ∼23.5 kDa, respectively). Both SUMOylation and mono-ubiquitination are critical, non-degradative signalling modifications that regulate synaptic protein function. A third possibility is the formation of covalent, non-disulphide dityrosine cross-links between NRGN monomers, a modification induced by oxidative stress and known to create stable oligomers of other synaptic proteins like tau.^45^

#### SYP, and SNAP25 (pre-synaptic proteins)

Synaptophysin (SYP) is the most abundant synaptic vesicle (SV) protein by mass, representing approximately 10% of total vesicle protein.^46^ A number of variable roles have been suggested for SYP in synaptic function including exocytosis, synapse formation, and endocytosis of SVs.^46^ One study observed that loss of SYP, resulted in defects in endocytosis.^46^ In our study, combining the cohort into three groups (control, PD/LBD-*GBA*, and PD/LBD-WT) to investigate mutant *GBA* groups and wild type groups, showed that in cingulate cortex, SYP was increased in wild type PD/LBD compared with controls but not with the *GBA* group. Although in our study the total *P*-value was significant, the pairwise *P*-value was almost significant. This could be due to a small N number. The trend of increased levels of SYP seen in both diseased groups in cingulate (the most affected area with α-syn load), suggesting that SYP might be needed to be overexpressed during this crucial stage of the disease due to its important and variable roles including exocytosis, and endocytosis.

Interestingly, both *GBA*, and WT groups showed a trend of SYP reduction in prefrontal and temporal cortices. Loss of SYP in these two brain regions could mean fewer synapses due to pre-synaptic loss, less SYP per synapse, or a mixture of both. It’s been reported that alteration of the various vesicular proteins can selectively change different forms of neurotransmitter releasee.^47^ Several studies show that non-synchronous forms of neurotransmitter release are essential to regulate synaptic plasticity, memory processing, and antidepressant action.^47–49^ Therefore, the possibility of selective loss of SYP protein to have functional effect on neurotransmission or vesicle recycling rather than synaptic loss cannot be neglected.

SNAP25 is an essential component of the SNARE complex which is fundamental to synaptic vesicle exocytosis. *SNAP25* gene has been associated with several distinct brain diseases, including attention deficit hyperactivity disorder (ADHD), schizophrenia, and bipolar disorder. This indicates that SNAP25 may act as a shared biological marker among several ‘synaptopathies’.^50^ Early-onset synaptic alterations were observed prior to DA neuronal loss in PD, therefore PD is also another synaptopathy,^51,52^ and our study confirms synaptic alteration in PD and other α-synucleinopathies. This study showed that SNAP25 was significantly reduced in PD/LBD-WT group compared with PD/LBD-*GBA* group in anterior cingulate, and temporal cortices whereas in prefrontal cortex SNAP25 was significantly increased in mutant PD/LBD-*GBA* group compared with both controls and PD/LBD-WT group. Furthermore, in parietal cortex, SNAP25 was significantly reduced in PD/LBD-WT group compared with controls. Despite the small number of PD/LBD-*GBA* cases, the difference found between *GBA* and WT groups was observed. This suggests that *GBA* may alter the pathway of certain markers, resulting in different disease progression pathway.

An important pattern of SNAP25 alteration was observed in our present investigations in several brain regions for each group representing recurrent increased levels of SNAP25 in PD/LBD-*GBA* group compared with both control, and PD/LBD-WT groups in three cortical regions (anterior cingulate, temporal, and prefrontal). Conversely, PD/LBD-WT group shows reduced SNAP25 levels compared with controls, and PD/LBD-*GBA* groups in three cortical regions (anterior cingulate, temporal and parietal). This may indicate that even though these two diseases have very similar diagnosis, but the mechanism underlying PD/LBD pathogenesis with and without *GBA* mutations, could be differently developed at the molecular level. PD/LBD associated with *GBA* mutations is known to be more severe, and invasive as the disease can be presented at earlier age of onset, and dementia can be developed faster. Looking closely to the graphs we observe that PD/LBD associated with N370S have higher pattern of SNAP25 levels compared with WT groups throughout the four regions with the most obvious in prefrontal cortex. This result suggests that the higher α-syn load previously reported in PD-associated *GBA* mutation may impede the necessary exocytosis function of SNAP25 in α-synucleinopathies. Thus, a higher expression of SNAP25 is observed in PD/LBD with *GBA* mutation in four cortical regions. Ultimately, a global reduction of both diseased groups was observed in the parietal cortex (the very late stage of α-syn pathology progression according to Braak staging system), suggesting that this reduction could be due to pre-synaptic loss, reduced SNAP25 levels, or a combination of both reasons.

### Three proteins in two cortical brain regions; (Rab3A, VAMP2 and VAMP3)/(anterior cingulate and prefrontal cortices)

#### Rab3A and VAMP2 (pre-synaptic proteins)

Rab3A is an abundant protein in SV. Rab3A forms a complex together with two another active zone proteins, and capture SVs for final membrane attachment called tethering. Exocytosis as well as neurotransmitter release were suggested to be altered in Lewy body diseases because α-syn aggregates were found to trap Rab3A, preventing it to interact with its binding partner, rabphilin (another pre-synaptic protein involved in exocytosis).^21,53^ In our study, Rab3A levels did not show any significant change between any of the three studied groups in either cortical region. Although, several recent studies in human, mouse and rat models presented a reduction in Rab3A,^14^ we don’t see significant change in our study, and a larger sample size may become more statistically meaningful.

Vesicle associated membrane proteins (VAMP) are a family of SNARE proteins known to share similar structure. The VAMP family are mostly involved in a number of steps of vesicular transport including vesicle fusion. VAMP2, known as synaptobrevin-2, is the most abundant and widely distributed VAMP protein in the brain. VAMP2 is essential for vesicular exocytosis and activity-dependent neurotransmitter release. VAMP2 was reduced in PD/LBD-*GBA,* and PD/LBD-WT groups compared with controls in both cortical brain regions. However, significant change was only seen in the late stage of the disease, prefrontal cortex, in both diseased groups compared with controls. Intriguingly, recent study reported that large α-syn oligomers bind preferentially to VAMP2, resulting in inhibition of vesicles docking.^54^ This could be the reason why cases with *GBA* mutation, the more severe diseased form with higher α-syn load, seems to have lower mean of VAMP2 levels compared with controls than WT group in both cortical regions.

#### VAMP3

VAMP3 is known as cellubrevin and is expressed in astrocytes. participates in regulation of exocytosis via Ca^2+^.^55–57^ Another study reported that VAMP3 vesicles go through Ca^2+^-independent exo- and endocytotic cycling at the plasma membrane of astrocytes.^58^ In the earlier, and more affected region with higher alpha syn load (cingulate cortex), VAMP3 was significantly increased in PD/LBD-*GBA* group compared with PD/LBD-WT. In contrast, VAMP3 was significantly reduced in the PD/LBD-WT group compared with the controls and PD/LBD-*GBA* groups. A similar pattern was displayed by SNAP25 in three cortical regions (anterior cingulate, temporal and prefrontal). One study reported that VAMP3 forms membrane fusion with the plasma membrane t-SNARE complexes, resulting in ternary complex including (syntaxin1/SNAP-25), (syntaxin1/SNAP-23) and (syntaxin4/SNAP-25).^59^ This event may explain why similar alteration profiles of proteins with similar functions (VAMP3, and SNAP-25) were observed. The significant increased level of VAMP3 in the PD/LBD-*GBA* group compared with the PD/LBD-WT group suggests that astrocytes could react differently in the *GBA* group than in the WT group. The increase of this marker in the PD/LBD-*GBA* may suggest the involvement of the astrocytes in this group associated with mutated *GBA*. This could be due to the increased α-syn inclusions in the most aggressive form of PD subtypes that was suggested by the three larger brain bank screens including studies from (NIH/University of Pennsylvania,^60^ Columbia University^61^ and the Queen Square Brain Bank^62^) which reported that *GBA* mutations were associated with widespread cortical LBs.

In the later stage of the disease, the prefrontal cortex, all diseased groups demonstrated a pattern of reduced VAMP3 levels compared with controls, suggesting neuronal, and/or glial cell loss.

It is important to acknowledge a limitation in our study concerning the specificity of VAMP3. We investigated VAMP3 not as a marker of astrocyte number, but as a functional marker of vesicular trafficking and exocytosis in astrocytes, based on its established roles in these pathways.^55,58^ However, given that VAMP3 is also expressed in other cell types, including neurons^63^ and microglia,^64^ the changes we observed cannot be definitively attributed solely to astrocytes. Instead, our findings likely represent a combined signal reflecting altered exocytotic function across multiple cell types. Future studies employing double-label immunohistochemistry to co-localize VAMP3 with specific astrocyte markers, such as GFAP or ALDH1L1, are warranted to dissect the precise cell-specific contributions to these changes.

### Correlations and confounding variables

The intricate network of protein-protein interactions at the synapse is fundamental to its function, and disruptions in this network are a hallmark of neurodegenerative disease. Our analysis of inter-protein correlations (Table 2) provides a snapshot of this synaptic architecture, revealing both preserved structural relationships and anomalous patterns that suggest a specific dysregulation of the presynaptic release machinery. A baseline of synaptic integrity is established by the consistent and statistically significant positive correlations between the canonical presynaptic vesicle protein Synaptophysin (SYP) and the postsynaptic scaffolding protein PSD95 in the anterior cingulate (BA24), temporal (BA21), and parietal (BA40) cortices. This strong pre-post synaptic coupling is a cornerstone of functional excitatory synapses, where vesicles containing SYP are targeted to the postsynaptic density, a structure heavily enriched with the scaffolding protein PSD95. The preservation of this fundamental relationship across multiple cortical regions serves as a crucial internal quality control, indicating that post-mortem degradation has not completely erased biologically meaningful protein networks.^65^ The notable exception is the frontal cortex (BA9), where this correlation was not significant, perhaps hinting at a region-specific vulnerability that warrants further investigation. Similarly, the strong positive correlation between the synaptic vesicle GTPase Rab3A and the v-SNARE VAMP2 further supports the integrity of the presynaptic terminal. Rab3A is essential for the activity-dependent transport and docking of synaptic vesicles at the active zone, while VAMP2 is a core component of the SNARE complex that executes membrane fusion.

Against this pattern of preserved synaptic machinery, SNAP25 was found to be negatively correlated with SYP and NRGN in BA24. SYP, VAMP2 and SNAP25 are all crucial presynaptic proteins that must cooperate to ensure efficient neurotransmitter release. SYP is known to form a complex with VAMP2, thereby regulating its trafficking and availability for SNARE complex assembly.^66^ VAMP2, in turn, forms the core four-helix bundle of the SNARE complex with SNAP25 and syntaxin to mediate vesicle fusion. This may reflect a state of pathological imbalance where the depletion or dysfunction of one component (e.g. SYP-VAMP2 complex) leads to a compensatory, but ultimately inefficient or mismatched, alteration in another (e.g. SNAP25).

Perimortem and post-mortem events can significantly alter the biochemical landscape of the brain. As expected, post-mortem delay (PMD) was negatively correlated with the levels of numerous proteins, most notably for the postsynaptic scaffolding protein PSD95 across all four brain regions, and for the presynaptic protein VAMP2 in BA24. This finding is consistent with a large body of literature demonstrating that the cessation of cellular homeostasis at death initiates enzymatic degradation (proteolysis), leading to a time-dependent decline in protein integrity and quantity.^67^ The particular susceptibility of PSD95 to PMD^68^ underscores accounting for this variable in quantitative analysis of post-mortem tissue. Interestingly, the relative robustness of SYP to PMD was also observed in the aforementioned study^68^ as well.

One striking finding from our analysis was a strong and consistent negative correlation between post-mortem tissue pH and the levels of the postsynaptic protein NRGN, particularly in BA9 and BA40, and to a lesser extent in BA24. This relationship, combined with the loss of significant group differences for NRGN after statistical adjustment for pH (Table 4), strongly suggests that a higher, less acidic pH environment observed in the PD/LBD N370S group is directly linked to lower measured levels of NRGN. The primary enzyme implicated in the cleavage of NRGN is Calpain-1, a calcium-activated neutral protease with optimal neutral pH.^69^ For example, m-calpain showed that its caseinolytic activity increased with increasing pH from 5.7 to 7.0.^70^ One potential explanation is that *GBA1* mutation can lead to lysosomal deacidification,^71^ metabolic compromise via mitochondrial dysfunction, and subsequently reduction in lactate metabolism during post-mortem hypoxia. This would result in less acidic post-mortem environment that allows the neutral protease especially calpain-1 to accelerate the degradation of NRGN. Even if the reduction in NRGN level results from the proposed post-mortem mechanisms, the changes in pH that contribute to this response are still *GBA1* mutation-dependent. The brief causal pathway would be: Disease → Ante-mortem Metabolic Dysfunction → Altered Post-mortem Acidification (Higher pH) → Increased Calpain-1 Activity → Lower NRGN Levels. Furthermore, the number of samples with available pH data is relatively small. Therefore, dismissing any potential antemortem disease effects on NRGN and presenting only the data after adjustment for pH would be statistically considered as over adjustment bias.

Conversely, positive correlation between pH and SNAP25 can be explained by inefficient degradation via lysosomal dysfunction and deacidification, which result in accumulation of SNAP25 in the terminals.

### Overall study and perspectives

A crucial point of discussion is whether the synaptic alterations observed in our cohort are a direct consequence of the *GBA* mutation or secondary to the extensive alpha-synuclein pathology. One possibility is a direct causal link, as the lysosomal dysfunction resulting from reduced GCase activity is known to disrupt essential cellular processes, including lipid metabolism and protein trafficking, that are vital for synaptic integrity.^72^ Alternatively, the synaptic deficits may be a downstream consequence of the severe alpha-synuclein pathology burden, which is a well-established driver of synaptic toxicity and dysfunction in synucleinopathies.^73^ However, the most plausible explanation is a synergistic and bidirectional relationship between the two. A compelling ‘two-hit’ model suggests that the genetic vulnerability from the *GBA* mutation initiates early synaptic and lysosomal deficits, which in turn accelerates the aggregation and propagation of alpha-synuclein. This accumulated pathology then further impairs lysosomal function, creating a vicious cycle that amplifies synaptic damage over the long course of the disease.^74^

While the conventional sample size calculation focuses on how many samples are required to yield statistical significance given assumed or preliminary effect sizes, we deem that an alternative estimation of how many samples are required to precisely reflect population standard deviation is of importance since it does not rely on prior measurement. Given a conventional threshold of acceptable coefficient of variation (CV) to be 30%, the goal is to find the minimum sample size such that the CV of sample SD is within 30% of the population SD. Under assumption of chi-square (*χ*^2^) distribution and normally distributed population, the CV of SD would be 28.20% for a sample size of 7, which is the sample size of the smallest group of this study. Based on empirical power analysis, among the variables showing the observed significant differences, NRGN BA24, SNAP25 BA21 and VAMP2 BA, showed relatively low statistical power when group sample sizes are equally at 7 while all other variables showed acceptable power of at least 70%. On the other hand, larger sample sizes would accentuate the difference for SYP in all regions except BA21 and Rab3A. Notably, BA21 showed small effect sizes for all proteins except SNAP25. A resting-state fMRI study provided evidence of higher regional homogeneity in the BA21 than other areas in DLB patients.^75^ Two possible explanations for elevation of SNAP25 in PD/LBD N370S can be as follows. Unlike VAMP2 or SYP that have transmembrane domain, SNAP25 is anchored to the membrane via palmitoylated cysteines^76^ such that impairment in glucosylceramide degradation can lead to over-stabilization of SNAP25 on lipid rafts.^77^ Endocytosis and vesicle-recycling proteins are downregulated in N370S *GBA* mutant and this can lead to accumulation of SNAP25 which originally reside on the membrane but depletion of vesicle-associated proteins such as VAMP2 from the pool.^78^ Although the study sample size was small, we observed a significant result in specific region with certain synaptic proteins. Therefore, the effect is not general, but rather potentially region-specific. Apart from the seven synaptic markers studied in this work, there are more than 1000 synaptic markers which have crucial functions in the disease progression and, therefore, conducting further studies to deeply understand complexity of disease progression and identify crucial and optimal time windows for targeted intervention.

One limitation of our study is the predominantly male composition of our cohorts, which precluded our ability to match cases or stratify statistical analyses by sex. This imbalance is, in part, a reflection of the known epidemiology of Parkinson's disease, which shows a higher incidence and progression to cognitive decline in males.^79^ Furthermore, our study was constrained by the limited availability of post-mortem tissue from individuals with these rare mutation cases within brain bank collections.

The clinical presentation of *GBA*-PD/LBD may involve an earlier age of onset and a more rapid progression (3 in 5 of N370S; 3 in 6 of L444P).^80^ Nonetheless, none of our N370S mutant had early age of onset with only one exception of aggressive disease progression (onset = 58, death = 66). There was one outlier early onset of age 50 but without *GBA1* mutation. One limitation of our cohorts is that an average age in the 80 s and average onset in the 60 s, represents a population with a very long disease duration and may not reflect the earlier pathological changes that drive disease onset and initial progression. In that regard, our study provides a valuable and rare window into the terminal, end-stage pathology of this specific genetic subtype. The data reveal the cumulative molecular changes present in the brain after decades of living with *GBA*-PD. These end-stage findings are crucial for understanding the ultimate consequences of the disease process, which may differ significantly from the initiating pathology.

Whole tissue homogenates instead of synaptoneurosomes were utilized for this study to provide a comprehensive overview of the total cellular protein content. This approach allows for the measurement of not only the mature vesicle pools at the synapse but also the presynaptic vesicle pools and postsynaptic protein that are newly synthesized within the soma before being transported to the terminals. Furthermore, all non-formalin-fixed brain tissues of this cohort were frozen per standard brain bank protocols and freezing can induce membrane breakdown and compartment leakage in the samples.^81^ Although it is conceptually feasible,^82^ it is quite difficult in practice to isolate synaptoneurosomes from frozen post-mortem brain tissue especially without specialized reagent e.g. Syn-PER™.^83^ This approach was selected not only to align with the study’s exploratory aims but also to address the significant practical challenges associated with isolating synaptically-enriched fractions from cryopreserved post-mortem tissue.

A key future direction is to correlate these synaptic alterations with measures of alpha-synuclein load under different chemical conditions in these cases together with the spatial quantification of various alpha-synuclein species with the immunohistochemistry (IHC) data as listed in the Supplementary Table 1.

Our work is consistent with several other studies and shows the importance of synaptic proteins in α-synucleinopathies. Our findings suggest that changed pre-synaptic, post-synaptic and astrocytic proteins, could all play a role in the pathogenesis of PD. More importantly, our *GBA* cohort (contain the largest collection of post-mortem human brain tissues with *GBA* mutations) demonstrates for the first time, to our knowledge, distinct expression patterns of specific synaptic markers in synucleinopathies linked to *GBA* mutations in post-mortem human tissue when compared with WT, suggesting that the molecular mechanisms underlying PD pathogenesis could be different in sporadic and genetic PD cohorts. These findings may explain why *GBA* carriers manifest early disease onset (early, mid–fifties), increased cognitive decline, and rapid disease progression compared with non-carriers.

The study shows that changes in synaptic and astrocytic proteins contribute to Parkinson’s disease (PD) pathogenesis, and for the first time demonstrates that *GBA* mutation carriers have distinct synaptic marker expression patterns, which may explain their earlier onset, faster progression, and greater cognitive decline compared with non-carriers.

The study reveals that GBA mutation carriers show distinct synaptic changes in Parkinson’s disease, explaining their earlier onset and faster progression.

## Supplementary Material

fcag246_Supplementary_Data

## Data Availability

The data that support the findings of this study are available from corresponding author upon request.
