# Supplementary material for: Altered synaptic and astrocytic proteins in Lewy body disorders associated with GBA mutations
Source: Brain Commun. 2026 Jul 23;8(4):fcag246. doi: 10.1093/braincomms/fcag246 (PMC13457929; doi:10.1093/braincomms/fcag246)
Supplement: Supplementary file 1 [file fcag246_Supplementary_Data.docx]

# Supplementary Materials

**Supplementary Table.1** The demographic data of each case in the cohort

| **Demographic Data** | | | | | | | | | | | | | |
| --- | --- | --- | --- | --- | --- | --- | --- | --- | --- | --- | --- | --- | --- |
| **Genotype** | **Deme-ntia** | **Brain Bank Source** | **Diag-nosis** | **Case No.** | **Code No.** | **Used in WB studies?** | **Used in IHC study?** | **Gender** | **Age at Death (yrs)** | **Age of Onset (yrs)** | **Disease Duration (yrs)** | **PMD (hrs)** | **pH** |
| *GBA*-N370S | Yes | UCL-QSBB | PDD | 1 | P25/13 | Yes | Yes | Male | 81 | 57 | 24 | 38 | N/A |
|  |  | Bristol-SWDBB | DLB | 2 | 359 | Yes | Yes | Male | 79 | 76 | 3 | 115 | 6.28 |
|  |  |  |  | 3 | 406 | Yes | Yes | Male | 66 | 58 | 8 | 23 | 6.13 |
| *GBA*-N370S | No | UCL-QSBB | PD | 4 | P31/00 | Yes | Yes | Male | 80 | 69 | 11 | 22 | 6.31 |
|  |  |  |  | 5 | P54/99 | Yes | Yes | Male | 92 | 79 | 13 | 60 | 6.22 |
|  |  |  |  | 6 | P31/03 | Yes | Yes | Male | 82 | 68 | 14 | 55 | N/A |
|  |  |  |  | 7 | P25/01 | Yes | Yes | Male | 85 | 63 | 22 | 49 | 6.38 |
| Wild-type (No *GBA* mutation) | Yes | UCL-QSBB | PDD | 8 | P13/97 | Yes | Yes | Male | 76 | 59 | 17 | 96 | N/A |
|  |  |  |  | 9 | P24/14 | Yes | No | Male | 92 | 77 | 15 | 42 | N/A |
|  |  |  |  | 10 | P65/15 | Yes | Yes | Male | 81 | 61 | 20 | 67 | 5.8 |
|  |  |  |  | 11 | P28/11 | Yes | No | Female | 86 | 66 | 20 | 64 | N/A |
|  |  |  |  | 12 | P9/08 | Yes | No | Male | 82 | 60 | 22 | 24 | N/A |
|  |  |  |  | 13 | P68/10 | Yes | Yes | Male | 73 | 50 | 23 | 43 | N/A |
|  |  |  | DLB | 14 | P13/09 | Yes | Yes | Male | 80 | 71 | 9 | 109 | 6.26 |
|  |  |  |  | 15 | P21/13 | Yes | No | Male | 82 | 64 | 18 | 33 | N/A |
|  |  | Bristol-SWDBB |  | 16 | 817 | Yes | No | Male | 89 | 88 | 1 | 40 | 6.08 |
|  |  |  |  | 17 | 867 | Yes | No | Male | 81 | 63 | 18 | 9 | 6.01 |
|  |  |  |  | 18 | 901 | Yes | No | Male | 94 | 94 | 0.5 | 32 | 6.02 |
| Wild-type (No *GBA* mutation) | No | UCL-QSBB | PD | 19 | P22/08 | Yes | No | Female | 80 | 71 | 9 | 48 | N/A |
|  |  |  |  | 20 | P25/04 | Yes | Yes | Male | 88 | 69 | 19 | 82 | N/A |
|  |  |  |  | 21 | P28/12 | Yes | No | Female | 82 | 61 | 21 | 136 | N/A |
|  |  |  |  | 22 | P38/04 | Yes | No | Female | 88 | 74 | 14 | 82 | N/A |
|  |  |  |  | 23 | P86/08 | Yes | No | Male | 81 | 74 | 7 | 37 | N/A |
|  |  |  |  | 24 | P80/11 | Yes | Yes | Male | 80 | 68 | 12 | 101 | N/A |
|  |  |  |  | 25 | P54/11 | Yes | Yes | Male | 81 | 62 | 19 | 67 | N/A |
|  |  |  |  | 26 | P20/10 | Yes | Yes | Male | 89 | 81 | 8 | 61 | 6 |
|  |  |  |  | 27 | P52/97 | Yes | Yes | Male | 85 | 63 | 22 | 34 | N/A |
| No *GBA* mutation | No | UCL-QSBB | Control | 28 | P75/08 | Yes | Yes | Male | 88 | N/A | N/A | 27 | 5.95 |
|  |  |  |  | 29 | P43/13 | Yes | No | Male | 88 | N/A | N/A | 97 | N/A |
|  |  |  |  | 30 | P48/15 | Yes | Yes | Male | 85 | N/A | N/A | 79 | 5.9 |
|  |  |  |  | 31 | P17/16 | Yes | No | Female | 84 | N/A | N/A | 54 | 5.61 |
|  |  |  |  | 32 | P72/07 | Yes | No | Male | 85 | N/A | N/A | 78 | 6.3 |
|  |  |  |  | 33 | P58/10 | Yes | No | Female | 88 | N/A | N/A | 52 | N/A |
|  |  |  |  | 34 | P75/10 | Yes | Yes | Male | 83 | N/A | N/A | 63 | N/A |
|  |  |  |  | 35 | P66/10 | Yes | No | Male | 87 | N/A | N/A | 57 | N/A |
|  |  | Bristol-SWDBB |  | 36 | 929 | Yes | No | Male | 81 | N/A | N/A | 36 | 6.04 |
|  |  |  |  | 37 | 766 | Yes | No | Male | 92 | N/A | N/A | 34 | 6.06 |

The code number describes a random number given to each case to mask the original Brain Bank case number for additional protection.

Cases with underlined code were from Bristol-SWDBB.

**Supplementary Table.2**

List of primary and secondary antibodies used for western blotting.

| Antibody /  RRID | Species/ clonality | Dilution /  Conc. | Supplier /  Cat No. | Secondary antibody |
| --- | --- | --- | --- | --- |
| β-tubulin  AB_2210370 | Rabbit polyclonal | 1:10,000  1 mg/ml | Abcam  ab6046 | IRDye 750 goat anti-Rabbit IgG |
| PSD-95  AB_1310620 | Rabbit monoclonal | 1:2500  NA | Abcam  ab76115 | IRDye 750 goat anti-Rabbit IgG  Rabbit |
| Neurogranin AB_447526 | Rabbit polyclonal | 1:2500  NA | Abcam  ab23570 | IRDye 750 goat anti-Rabbit IgG  Rabbit |
| SNAP-25 AB_887794 | Mouse monoclonal | 1:5000  1 mg/ml | Synaptic systems  # 111 011 | IRDye 680 goat anti-Mouse IgG  Mouse |
| Synaptophysin AB_2198854 | Mouse monoclonal | 1:3000  1 mg/ml | Abcam  ab8049 | IRDye 680 goat anti-Mouse IgG  Mouse |
| Rab3A AB_887769 | Rabbit polyclonal | 1:5000  1 mg/ml | Synaptic systems  # 107 102 | IRDye 750 goat anti-Rabbit IgG  Rabbit |
| VAMP3/2*  AB_10001199 | Rabbit polyclonal | 1:1250  1 mg/ml | Novus biologicals  NB300-515 | IRDye 750 goat anti-Rabbit IgG  Rabbit |

VAMP3/2* is a VAMP3 antibody that can recognise the epitope of VAMP2 as well. This antibody has been tested and confirmed to be able to recognise both VAMPs 3 and 2.

**Supplementary Fig.1** Scatter plot for PSD-95 over β-tubulin in four brain regions and between three groups. **(A, B, C, D)** PSD-95 levels in anterior cingulate, temporal, prefrontal, and parietal cortices respectively. KW test was performed on all brain regions with the effect sizes η²_H_ of 0.023, 0.000, 0.000, and 0.000 respectively. Black dots signify cognitively healthy control, whereas red and black triangle signify PD/LBD with *GBA1* N370S mutation and *GBA1* WT respectively. Bars represent mean ± SEM. * *p*<0.05, ** *p*<0.01, *** *p*<0.001, and *****p*<0.0001. The presented images are example of representative blots, and the full images of WB membranes are shown in the Supplementary Materials with the corresponding positional codes of the representative bands in the full membrane (letter as a membrane or row ID and number as column ID) are as follows:

**A =** Supplementary Fig.4 [BR,E3,E4,A1,A5,C1,C3], **B =** Supplementary Fig.5 [ER,E3,E4,A1,A5,B8,D5],

**C =** Supplementary Fig.6 [BR,E3,E4,A1,A5,B3,B8], **D** = Supplementary Fig.7 [AR,E3,E4,A1,A5,B4,D3].

**Supplementary Fig.2** Scatter plot for synaptophysin (SYP) over β-tubulin in four brain regions, and between three groups. **(A, B, C, D)** SYP levels in anterior cingulate, temporal, prefrontal, and parietal cortices respectively. KW test was performed on all of the brain region with the effect sizes η²_H_ of 0.126, 0.002, 0.065, and 0.070 respectively. Black dots signify cognitively healthy control, whereas red and black triangle signify PD/LBD with *GBA1* N370S mutation and *GBA1* WT respectively. Bars represent mean ± SEM. * *p*<0.05, ** *p*<0.01, *** *p*<0.001, and *****p*<0.0001. The presented images are example of representative blots, and the full images of WB membranes are shown in the Supplementary Materials with the corresponding positional codes of the representative bands in the full membrane (letter as a membrane or row ID and number as column ID) are as follows:

**A =** Supplementary Fig.4 [BR,E3,E4,A1,A5,C1,C3], **B =** Supplementary Fig.5 [ER,E3,E4,A1,A5,B8,D5],

**C =** Supplementary Fig.6 [BR,E3,E4,A1,A5,B3,B8], **D** = Supplementary Fig.7 [AR,E3,E4,A1,A5,B4,D3].

**Supplementary Fig.3** Scatter plot for Rab3A over β-tubulin in two cortical brain regions and between three groups. **(A, B)** Rab3A levels in anterior cingulate, and prefrontal cortices. KW test was performed on both cortical brain regions with the effect sizes η²_H_ of 0.048 and 0.037 respectively. Bars represent mean ± SEM. * *p*<0.05, ** *p*<0.01, *** *p*<0.001, and *****p*<0.0001. The presented images are example of representative blots, and the full images of WB membranes are shown in the Supplementary Materials with the corresponding positional codes of the representative bands in the full membrane (letter as a membrane or row ID and number as column ID) are as follows:

**A =** Supplementary Fig.12 [ER,E1,E2,A1,A4,C5,D2], **B =** Supplementary Fig.13 [CR,E3,E4,A3,A5,B5,C6],

### Full representative Western blot membranes for synaptic markers

**Supplementary Fig.4** Representative individual set of all cases from the triplicate for SYP, PSD95, and β-tubulin in the anterior cingulate cortex (BA24). Membrane pictures in the same row were from the same membrane. R is for rat cortex.

**Supplementary Fig.5** Representative individual set of all cases from the triplicate for SYP, PSD95, and β-tubulin in the temporal cortex (BA21). Membrane pictures in the same row were from the same membrane. R is for rat cortex.

**Supplementary Fig.6** Representative individual set of all cases from the triplicate for SYP, PSD95, and β-tubulin in the prefrontal cortex (BA9). Membrane pictures in the same row were from the same membrane. R is for rat cortex.

**Supplementary Fig.7** Representative individual set of all cases from the triplicate for SYP, PSD95, and β-tubulin in the parietal cortex (BA40). Membrane pictures in the same row were from the same membrane. R is for rat cortex.

**Supplementary Fig.8** Representative individual set of all cases from the triplicate for NRGN, SNAP25, and β-tubulin in the anterior cingulate cortex (BA24). Membrane pictures in the same row were from the same membrane. R is for rat cortex.

**Supplementary Fig.9** Representative individual set of all cases from the triplicate for NRGN, SNAP25, and β-tubulin in the temporal cortex (BA21). Membrane pictures in the same row were from the same membrane. R is for rat cortex.

**Supplementary Fig.10** Representative individual set of all cases from the triplicate for NRGN, SNAP25, and β-tubulin in the prefrontal cortex (BA9). Membrane pictures in the same row were from the same membrane. R is for rat cortex.

**Supplementary Fig.11** Representative individual set of all cases from the triplicate for NRGN, SNAP25, and β-tubulin in the parietal cortex (BA40). Membrane pictures in the same row were from the same membrane. R is for rat cortex.

**Supplementary Fig.12** Representative individual set of all cases from the triplicate for Rab3A, VAMP2, VAMP3 and β-tubulin in the anterior cingulate cortex (BA24). Membrane pictures in the same row were from the same membrane. R is for rat cortex.

**Supplementary Fig.13** Representative individual set of all cases from the triplicate for Rab3A, VAMP2, VAMP3 and β-tubulin in the prefrontal cortex (BA9). Membrane pictures in the same row were from the same membrane. R is for rat cortex.

**Supplementary Fig.14** Heat map showing empirical statistical powers for most significant pairwise difference of each variable calculated by resampling of two given groups at varying equal sample sizes. On that note, resampling at sample size used in the experiment (control = 10, PD/LBD N370S = 7, PD/LBD WT = 20) was also conducted as reference. Lastly, Cohen’s *d* effect sizes were calculated from the statistical powers given varying sample sizes and expressed as Median ± MAD.
